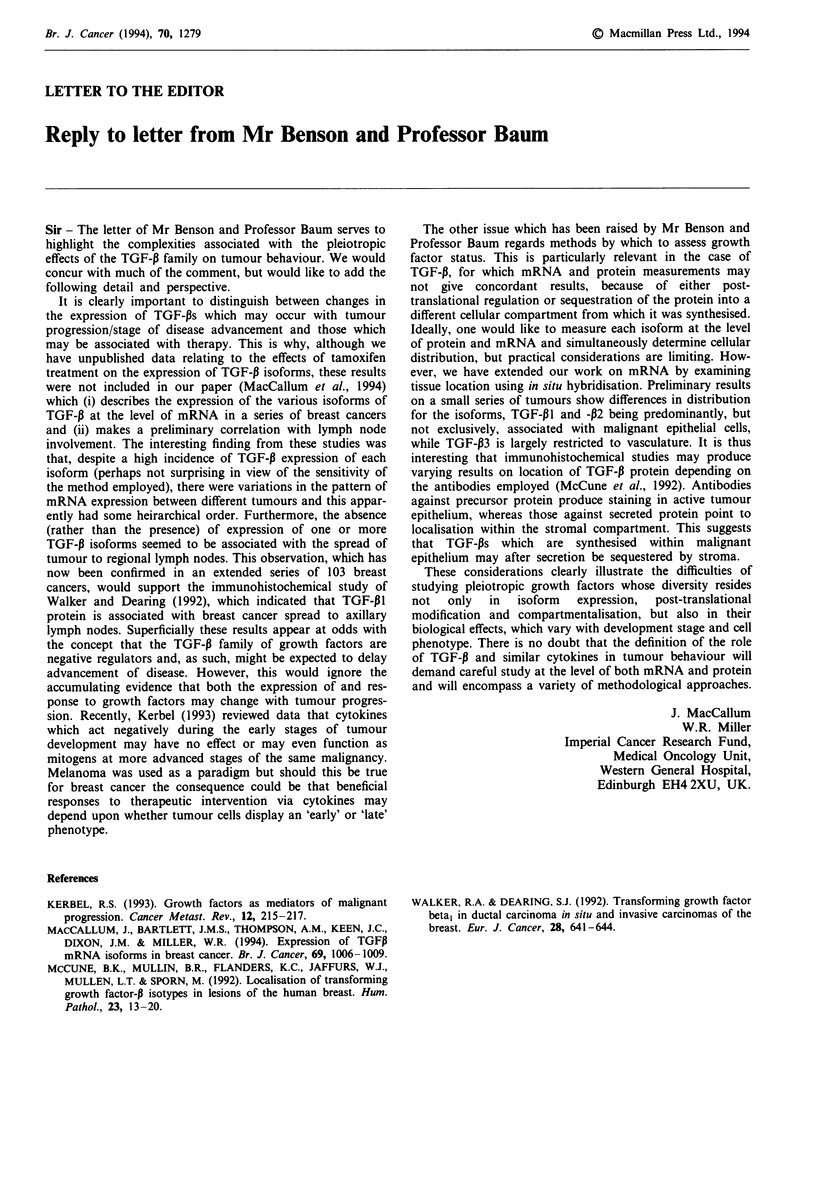# Reply to letter from Mr Benson and Professor Baum

**Published:** 1994-12

**Authors:** J. MacCallum, W.R. Miller


 © Macmillan Press Ltd., 1994

LETTER TO THE EDITOR

### Reply to letter from Mr Benson and Professor Baum

**Sir** – The letter of Mr Benson and Professor Baum serves to highlight the complexities associated with the pleiotropic effects of the TGF-β family on tumour behaviour. We would concur with much of the comment, but would like to add the following detail and perspective.

It is clearly important to distinguish between changes in the expression of TGF-βs which may occur with tumour progression/stage of disease advancement and those which may be associated with therapy. This is why, although we have unpublished data relating to the effects of tamoxifen treatment on the expression of TGF-β isoforms, these results were not included in our paper (MacCallum *et al.*, 1994) which (i) describes the expression of the various isoforms of TGF-β at the level of mRNA in a series of breast cancers and (ii) makes a preliminary correlation with lymph node involvement. The interesting finding from these studies was that, despite a high incidence of TGF-β expression of each isoform (perhaps not surprising in view of the sensitivity of the method employed), there were variations in the pattern of mRNA expression between different tumours and this apparently had some heirarchical order. Furthermore, the absence (rather than the presence) of expression of one or more TGF-β isoforms seemed to be associated with the spread of tumour to regional lymph nodes. This observation, which has now been confirmed in an extended series of 103 breast cancers, would support the immunohistochemical study of Walker and Dearing (1992), which indicated that TGF-β1 protein is associated with breast cancer spread to axillary lymph nodes. Superficially these results appear at odds with the concept that the TGF-β family of growth factors are negative regulators and, as such, might be expected to delay advancement of disease. However, this would ignore the accumulating evidence that both the expression of and response to growth factors may change with tumour progression. Recently, Kerbel (1993) reviewed data that cytokines which act negatively during the early stages of tumour development may have no effect or may even function as mitogens at more advanced stages of the same malignancy. Melanoma was used as a paradigm but should this be true for breast cancer the consequence could be that beneficial responses to therapeutic intervention via cytokines may depend upon whether tumour cells display an 'early' or 'late' phenotype.

The other issue which has been raised by Mr Benson and Professor Baum regards methods by which to assess growth factor status. This is particularly relevant in the case of TGF-β, for which mRNA and protein measurements may not give concordant results, because of either post-translational regulation or sequestration of the protein into a different cellular compartment from which it was synthesised. Ideally, one would like to measure each isoform at the level of protein and mRNA and simultaneously determine cellular distribution, but practical considerations are limiting. However, we have extended our work on mRNA by examining tissue location using *in situ* hybridisation. Preliminary results on a small series of tumours show differences in distribution for the isoforms, TGF-β1 and -β2 being predominantly, but not exclusively, associated with malignant epithelial cells, while TGF-β3 is largely restricted to vasculature. It is thus interesting that immunohistochemical studies may produce varying results on location of TGF-β protein depending on the antibodies employed (McCune *et al.*, 1992). Antibodies against precursor protein produce staining in active tumour epithelium, whereas those against secreted protein point to localisation within the stromal compartment. This suggests that TGF-βs which are synthesised within malignant epithelium may after secretion be sequestered by stroma.

These considerations clearly illustrate the difficulties of studying pleiotropic growth factors whose diversity resides not only in isoform expression, post-translational modification and compartmentalisation, but also in their biological effects, which vary with development stage and cell phenotype. There is no doubt that the definition of the role of TGF-β and similar cytokines in tumour behaviour will demand careful study at the level of both mRNA and protein and will encompass a variety of methodological approaches.

J. MacCallum
W.R. Miller
Imperial Cancer Research Fund,
Medical Oncology Unit,
Western General Hospital,
Edinburgh EH4 2XU, UK.

### References

KERBEL, R.S. (1993). Growth factors as mediators of malignant progression. *Cancer Metast. Rev.*, **12**, 215–217.

MACCALLUM, J., BARTLETT, J.M.S., THOMPSON, A.M., KEEN, J.C., DIXON, J.M. & MILLER, W.R. (1994). Expression of TGFβ mRNA isoforms in breast cancer. *Br. J. Cancer*, **69**, 1006–1009.

MCCUNE, B.K., MULLIN, B.R., FLANDERS, K.C., JAFFURS, W.J., MULLEN, L.T. & SPORN, M. (1992). Localisation of transforming growth factor-β isotypes in lesions of the human breast. *Hum. Pathol.*, **23**, 13–20.

WALKER, R.A. & DEARING, S.J. (1992). Transforming growth factor $beta_1$ in ductal carcinoma *in situ* and invasive carcinomas of the breast. *Eur. J. Cancer*, **28**, 641–644.